# Exploring the perspectives and strategies of Ontario community pharmacists to improve routine follow-up for patients with diabetes: A qualitative study

**DOI:** 10.1177/17151635211018479

**Published:** 2021-06-30

**Authors:** Natali Surkic, Annalise Mathers, Jamie Kellar, Lori MacCallum, Lisa Dolovich

**Affiliations:** Leslie Dan Faculty of Pharmacy, University of Toronto; Leslie Dan Faculty of Pharmacy, University of Toronto; Leslie Dan Faculty of Pharmacy, University of Toronto; Leslie Dan Faculty of Pharmacy, University of Toronto; Banting and Best Diabetes Center, Toronto; Leslie Dan Faculty of Pharmacy, University of Toronto; Department of Family Medicine, McMaster University, Hamilton; School of Pharmacy, University of Waterloo, Waterloo, Ontario

## Abstract

**Background:**

Medication reviews are a fundamental activity carried out as part of comprehensive care delivered by pharmacists. Varying programs that reimburse pharmacists for conduct of medication reviews are in place in different jurisdictions in Canada and other countries around the world. The MedsCheck Diabetes (MCD) program is a publicly funded service in Ontario, Canada, offered to patients with type 1 or type 2 diabetes. Through this service, pharmacists can complete a focused medication review with advice, training, monitoring and follow-up diabetes education. Although pharmacists can be reimbursed for patient follow-up activities, a low number of follow-up medication reviews are billed through this program.

**Methods:**

The study explores the barriers and facilitators that community pharmacists in Ontario experience in conducting routine monitoring and follow-up of patients with diabetes. Using a descriptive content analysis approach study, semistructured interviews were conducted with a convenience sample of 8 community pharmacists working in Ontario.

**Results:**

Three main themes emerged: the design of the MCD program, the state of community pharmacy and collaboration and relationships. These themes demonstrate challenges and potential strategies recognized by community pharmacists to conduct routine diabetes follow-up through the MCD program.

**Conclusion:**

This study found that the design of the MedsCheck Diabetes program, the community pharmacy environment and the relationships between pharmacists, patients and prescribers can pose a challenge in the conduct of routine monitoring and follow-up through the MedsCheck Diabetes program.

**Knowledge Into Practice**

- Ontario-based community pharmacists can be reimbursed for patient follow-up through the publicly funded MedsCheck Diabetes (MCD) program; however, a low number of follow-up medication reviews are billed through this program.
- This study explored perspectives of community pharmacists in Ontario on barriers and facilitators to routine monitoring and follow-up of patients with diabetes.
- Key themes illustrated that the design of the MCD program, the state of community pharmacy and collaboration and relationships are important factors in helping or hindering the conduct of routine follow-up through the MCD program.
- Changes to the MCD program could improve the ability of community pharmacies to provide proactive follow-up care that fosters collaboration and strengthens relationships both within the pharmacy, with patients and with external health care providers.

**Mise En Pratique Des Connaissances**

- Les pharmaciens de l’Ontario peuvent être remboursés pour le suivi des patients dans le cadre du programme MedsCheck Diabetes (MCD), financé par l’État; toutefois, un faible nombre de vérifications de suivi de médicaments sont facturées dans le cadre de ce programme.
- Cette étude a essayé d’avoir les points de vue des pharmaciens de l’Ontario sur les obstacles et les facteurs facilitant la surveillance et le suivi de routine des patients atteints de diabète.
- Les principaux thèmes ont démontré que la conception du programme MCD, l’état de la pharmacie communautaire, ainsi que la collaboration et les relations sont des facteurs importants pour aider ou entraver la réalisation d’un suivi de routine par le biais du programme MCD.
- Les modifications apportées au programme MCD pourraient améliorer la capacité des pharmacies communautaires à fournir des soins de suivi proactifs qui favorisent la collaboration et renforcent les relations au sein de la pharmacie, avec les patients et avec les prestataires de soins de santé externes.

## Background

Almost 10% of the Canadian population lives with diabetes.^1^ Diabetes is a chronic disease that causes many potentially debilitating and long-term complications. People living with diabetes are over 3 times more likely to be hospitalized with cardiovascular disease, 12 times more likely to be hospitalized with end-stage renal disease and over 20 times more likely to be hospitalized for nontraumatic lower limb amputations compared to the general population.^1^ Moreover, 50% of people living with diabetes in Canada are not achieving their blood glucose targets, 64% are not achieving blood pressure targets and 43% are not achieving lipid targets.^2^

The collaboration of many health care professionals, including pharmacists, can improve diabetes care through the provision of patient education and the planning and implementation of holistic care plans.^3^ Community pharmacists are in a unique position to contribute to diabetes management due to their knowledge of and expertise in medication management,^4^ as well as being broadly accessible in large or small, rural or urban communities in Canada and most countries worldwide.^4,5^ Active involvement of a community pharmacist in the management of patients’ chronic diseases, such as diabetes, has been shown to optimize medication management and patient outcomes.^6-8^

One opportunity community pharmacists in Ontario have available to facilitate care provision for people with diabetes is through the MedsCheck Diabetes (MCD) program. A MCD service is provided to patients with type 1 or type 2 diabetes and includes a focused medication review with advice, training, monitoring and education on diabetes.^9^ Anyone with a valid Ontario health card and who has been diagnosed with diabetes is eligible for an MCD. Pharmacists are reimbursed $75 per patient for an initial MCD appointment that can be done annually and $25 per patient for a follow-up appointment.^9^ There is no limit on the number of follow-up appointments that can be billed. MCD services cannot be conducted over the phone or through videoconferencing, and the follow-up must take place at the same pharmacy that provided the initial service.^10^ The program was designed to promote continuity of care for the patient so that the initial appointment can guide subsequent medication management. A similar medication management program is also in place in Australia.^11^

Previous research has demonstrated that only about 2.7% to 4.1% of patients who received an MCD also received follow-up by their community pharmacist through the MCD program.^12^ While literature has broadly examined the community pharmacist’s role in diabetes care,^13^ there is benefit to increasing the depth of understanding regarding community pharmacists’ roles, barriers and facilitators in conducting follow-up after an initial medication review for a patient with diabetes.

The objective of this study was to explore perspectives of community pharmacists in Ontario on barriers and facilitators to routine monitoring and follow-up of patients with diabetes. This was the first phase of a multiphase study, and the findings reported here were used to help develop an online survey administered to pharmacists across Canada.^13^

## Methods

Study participants included community pharmacists working in Ontario who had a Part A (active) license and experience providing MedsChecks to people with diabetes. Participants were recruited through a convenience sample approach, using existing investigator networks, but there was no existing relationship between the interviewer and the participants.

The interviewer contacted the participants to provide an introduction to the study and set up a time for the interview. Participants were aware that the interviewer was part of the research team and a Doctor of Pharmacy student at the University of Toronto.

One-on-one interviews with community pharmacist participants were conducted using a 5-question semistructured interview guide (Appendix 1, available online at www.cpjournal.ca). Interviews were conducted over the phone and were approximately 30 minutes in length. The interviewer was female and received training and conducted pilot testing with a researcher experienced in qualitative interviewing.

All interviews were audiotaped, transcribed verbatim, deidentified and cleaned. No field notes were taken during the interviews. Interviews were conducted until saturation occurred, when identified responses from interview participants began to repeat and no new themes were emerging from the interviews.^14^

We used a qualitative descriptive content analysis approach,^15^ which is a systematic method of analyzing data to categorize and understand the meaning of trends and patterns in the data based on descriptions and characteristics of the research content itself. Three research team members individually and manually analyzed each interview transcript. The researchers identified themes from each interview and then compared individual findings to triangulate the data collected.^16^ These themes were synthesized into a master document. Common themes were identified and reviewed by the research team over multiple discussions, leading to further refinement of theme and subtheme triangulation. Each quote provided below has an associated number in parentheses denoting the study participant number.

The study was approved by the Research Ethics Boards at the University Health Network and the University of Toronto.

## Results

Eight pharmacist participants were interviewed in this study. Participants had a mean age of 44 years old (minimum-maximum, 27-52 years). Four participants practised in independent pharmacies, 2 in banner pharmacies and 2 in franchise pharmacies. No participants dropped out, and each participant was interviewed once.

Three main thematic areas emerged that form the basis of the findings of our study: Design of the MedsCheck Program, Environment of Community Pharmacy and Collaboration and Relationships.

### Design of the MedsCheck program

Participants described that follow-up does occur regularly in community pharmacy but that these activities are often not channeled through the MCD program due to program requirements and the relative ease of following up with patients informally.

#### Criteria for MedsCheck Diabetes follow-up

Some participants felt that the requirement for the patient to be physically present for the MCD follow-up program was too restrictive. These participants reported that follow-up often occurs through various other means (which do not qualify for reimbursement): “Patients have my email and they have my cell phone and they will text me questions” (1).

#### Documentation

Most participants stated that although they regularly conducted routine monitoring and follow-up with their patients, they often did not have time to complete the documentation required by the MCD program due to other workload priorities. One participant expressed that “my biggest barrier right now to even getting to the point of doing a follow-up with diabetes would be the paperwork and time commitment” (5).

#### Diabetes follow-up integrated into holistic care

Although the majority of pharmacists stated that they do provide routine follow-up and monitoring, 1 participant described that “formally, we are not doing a diabetic follow-up commitment at all. That being said there is diabetic follow-up. There is follow-up to all disease states” (5). This comment emphasizes that although pharmacists have the intention to, and often do, follow up with patients, it is completed as part of routine patient care, rather than channeled through the MCD program.

### Environment of community pharmacy

The community pharmacy setting and setup were seen by participants as unfavourable to conducting follow-up through the MCD program. Workflow, staff and technology have to be available and coordinated to complete regular follow-up that is effective, efficient and financially viable within the pharmacy.

#### Pharmacy workflow

All pharmacists discussed pharmacy workflow as critical to the success of conducting follow-up with patients. Many participants stated that scheduling appointments with patients improved their ability to follow up, particularly when follow-up was scheduled immediately after the initial MedsCheck: “We’re starting to adjust his insulin and we’ve scheduled him back every 48 hours for a while” (7).

Participants discussed giving the patient a business card with the appointment details on it and entering the appointment into an online calendar at the pharmacy with a note outlining the purpose of the appointment for the pharmacy staff. Half the participants felt that scheduling an appointment helped, as “most people prefer to do the follow-up while they’re picking up their medication because they’re already in the pharmacy” (8). Participants expressed that a walk-in model was “a shot in the dark. Some days it worked and other days it didn’t” (3).

#### Technicians and front store staff

Half of the participants mentioned that investing in regulated pharmacy technicians alleviated workflow pressures on the pharmacist and allowed them to engage in more clinical services: “I have 3 registered techs working most days . . . the way we operate, I wouldn’t be able to do [clinical services] without them” (7).

One participant spoke to the significance of engaging front shop staff to promote clinical services, “because a lot of times they’re initiating it. They’re saying you know our pharmacist will sit down with you and talk to you about this and monitor you . . . it’s the entire staff that can really promote professional services” (7).

#### Pharmacy technologies

One participant said that many of their patients were lost to follow-up because their pharmacy doesn’t “have a specific system for following up with patients . . . when you have a complex patient you try to line them up and tackle one thing at a time and then you hope to call them up for a follow-up in a month or in 2 weeks depending on the case, but this usually falls through the cracks” (3).

Pharmacists who had electronic systems to easily retrieve existing MedsCheck documentation found that this reduced redundancy and ensured continuity with subsequent patient interactions. One participant said that “we scan all our documents into the patient file, it makes it a lot easier that the pharmacist can go into the patient profile, look at what was said in the last MedsCheck review and then continue from that point on so that you’re not wasting time asking the same questions and patients getting frustrated that they’ve already given you that information” (8). Some participants also said that their pharmacy software system allows them to set reminders for scheduled follow-up.

#### Economics of community pharmacy

One participant mentioned that the MCD program may only be viable for established pharmacy businesses: “There are times when I spend more than 30 or 45 minutes with a patient to do a follow-up and we get $25 from ODB . . . we’re lucky to have a steady business so that we can support activities like that” (6). Another participant added that follow-up is more of a patient retention strategy rather than a viable revenue source: “I don’t really look at MedsCheck as a financial revenue stream. MedsCheck itself I look at it as basically for patients—I want to make sure I’m developing a relationship with them that they’re going to be a customer long term where they feel comfortable with all their health care decisions” (7).

### Collaboration and relationships

Finally, the significance of collaboration and cultivating relationships with patients and physicians presented as key influencers of success of follow-up in the MCD program.

#### Patients

Some participants felt that many patients did not understand the clinical role of the pharmacist or understand the purpose of the MCD program: “A lot of times people don’t know this is part of our service. So, you know for me it’s being able to have the patients understand that we can book appointments, we can sit down one on one, and we can come to their house to do appointments” (7).

One participant stated that they had taken the initiative to educate their community on the pharmacist’s scope of practice, resulting in patients beginning to independently reach out to the pharmacy.

#### Physicians

Some participants stated that even when they invest substantial time into making a thorough recommendation, physicians are not always aware of pharmacists’ scope of practice. One participant said that after faxing a recommendation, “I had one physician respond to me and in a very angry manner because I had questioned him . . . he called me the next day asking me what’s a MedsCheck? Why are you doing this? Why are you making interventions?” (4).

Some participants mentioned that they have good working relationships with the physicians involved in their patients’ care. One of these participants commented that a significant number of their follow-ups occurred through physician referral: “It took us a few years, to establish my expertise and reputation . . . I’ve been building up my expertise and talking to prescribers” (6).

## Discussion

This study identifies that there is poor alignment between the specifications of the MCD program and how it can be implemented in the community pharmacy setting. Although community pharmacists report that they often provide routine follow-up to their patients with diabetes, these interactions are inconsistently channeled through the MCD program. Our findings suggest that this is due to the design of the MedsCheck Diabetes program, the community pharmacy environment and the current relationships between pharmacists, patients and physicians. Our findings show that while pharmacists understand and endorse their professional responsibility in providing comprehensive care to patients with diabetes and are equipped with the skills to actively conduct routine follow-up, a number of factors impede the success of the MCD program at this time.

Our research shows that community pharmacy workflow is not always conducive to offering MCD follow-up on an in-person walk-in basis. This may result in missed opportunities for follow-up; for documentation to be rushed, incomplete or of poor quality; and for billing for reimbursement to not occur. As our findings and previous research suggests, coordinating MCD appointments with patients coming into the pharmacy to pick up medication could blend the reliability of scheduled follow-up with the convenience of medication pick-up and integrate clinical services into pharmacy workflow.^17^ Virtual MedsCheck visits, which have been permitted during the COVID-19 pandemic, may also improve the implementation of follow-up visits since the logistical challenge of people travelling to the pharmacy is avoided.^18^

Additionally, a major barrier for pharmacists to conduct follow-up through the MCD program is the extensive documentation required. Pharmacist participants reported not always documenting follow-up activities using MCD-approved forms. This is consistent with other studies that have identified the need to employ automated documentation systems to reduce documentation barriers for MedsCheck services.^19^ This optimization is important because documentation of patient care activities has been shown to be crucial for continuity of care, justifying pharmacist salary and sharing information with other health care providers.^20^

Our study respondents felt the community pharmacy business model in Ontario was poorly aligned with the MCD program structure. These findings are consistent with other reports identifying that there is a balance between profitability and patient care experienced by many health care professionals; for example, family physicians and nurse practitioners in Ontario are compensated a standard fee for each service they provide.^21^ Ontario pharmacists are remunerated for MCD follow-up in a similar way, but the pharmacists interviewed in this study felt that the remuneration from the MCD program was not financially sustainable.^10^ This is consistent with the underlying need for revisions to the pharmacy business model to incorporate broader clinical care, including chronic disease prevention and management.^22,23^ Further research in this area would provide insight into alternative pharmacy care models that optimize the balance between patient health outcomes, workflow and profit.

Finally, our study points to the well-identified need to improve patient and physician understanding of the role of community pharmacists in diabetes management and awareness of the MCD program. Many patients are unaware of the services available at their community pharmacy, as well as the pharmacist’s role in their care.^13^ Lack of awareness among physicians regarding the MCD program may also present a major barrier, as the pharmacist may be unable to make clinical interventions without a physician’s authorization. Therefore, a weak relationship between pharmacists and physicians may have a profound impact on the pharmacist’s ability to follow up with their patients and, in turn, provide recommendations to improve the patient’s therapeutic regimen.^24,25^

The MCD program was designed as a tool for pharmacists to provide quality, effective follow-up for patients with diabetes. The MCD program should be a helpful tool, given the critical need for diabetes care.^1,9^ However, due to poor alignment between the program, community pharmacy and the broader health care environment, only about 2.7% to 4.1% of MCD patients receive follow-up by community pharmacists through the MCD program.^12^ This study suggests several mechanisms that could support the restructuring of the MCD program and workflow in community pharmacy to better enable pharmacists to conduct continuous and proactive follow-up care for people with diabetes through the MCD program (Table 1).

**Table 1:** Barriers and strategies associated with the MedsCheck Diabetes (MCD) program and community pharmacy

Barrier	Strategy
Patient must be physically present for the MCD follow-up	Redesign MCD program to include remuneration through various mediums (e.g., phone, email, video call, in person)
Extensive documentation and time required by the MCD program	Employ automated documentation systems to streamline documentation and retrieval of previously gathered information Invest in pharmacy technicians to allow pharmacists to focus more on clinical service provision
Walk-in model for MCD consultations is inconsistent for workflow and staff	Schedule follow-up appointments in advance Coordinate MCD follow-up appointments with medication pickup Provide patients with a reminder card with the appointment details
Pharmacy software does not have a system to track and follow up with patients	Use systems that can track and set scheduled appointment reminders for follow-up
Patients unaware of services they may be eligible to receive from their pharmacist, as well as clinical skills of the pharmacist	Initiate community education programs regarding the pharmacist’s scope of practice Engage front store staff to promote clinical services
Prescribers unaware of pharmacist scope of practice and the MCD program guidelines	Engaging with prescribers to raise awareness and enhance opportunities for collaboration

### Strengths and limitations

Pharmacists interviewed in this study were all familiar with the MCD program and strongly supported improvements in MCD follow-up in community pharmacies. However, limitations of this study include the small sample size of participants and the convenience sample used.

## Conclusion

This study found that the design of the MedsCheck Diabetes program, the community pharmacy environment and the relationships between pharmacists, patients and prescribers can pose a challenge in the conduct of routine monitoring and follow-up through the MedsCheck Diabetes program. Future research should aim to more closely examine and implement strategies to optimize routine monitoring and follow-up in community pharmacy for patients with diabetes, taking into consideration the themes identified in this study. ■

## Supplemental Material

sj-pdf-1-cph-10.1177_17151635211018479 – Supplemental material for Exploring the perspectives and strategies of Ontario community pharmacists to improve routine follow-up for patients with diabetes: A qualitative study Click here for additional data file. Supplemental material, sj-pdf-1-cph-10.1177_17151635211018479 for Exploring the perspectives and strategies of Ontario community pharmacists to improve routine follow-up for patients with diabetes: A qualitative study by Natali Surkic, Annalise Mathers, Jamie Kellar, Lori MacCallum and Lisa Dolovich in Canadian Pharmacists Journal / Revue des Pharmaciens du Canada
