# Supplementary material for: Exploring the perspectives and strategies of Ontario community pharmacists to improve routine follow-up for patients with diabetes: A qualitative study
Source: Can Pharm J (Ott). 2021 Jun 30;154(5):342–8. doi: 10.1177/17151635211018479 (PMC8408913; doi:10.1177/17151635211018479)
Supplement: sj-pdf-1-cph-10.1177_17151635211018479 – Supplemental material for Exploring the perspectives and strategies of Ontario community pharmacists to improve routine follow-up for patients with diabetes: A qualitative study [file sj-pdf-1-cph-10.1177_17151635211018479.pdf]

Appendix 1 Interview guide

Participant ID Code: _________ Date Completed: _________

Introduction (pre-audio recording):

Thank you for agreeing to participate in this interview. The interview is estimated to take 30 minutes. The purpose of the interview is to obtain your views on regular monitoring and follow up in your community pharmacy and your views on what helps and hinders those processes.

With your permission, I would like to audio-record this interview. The interview recording will be transcribed verbatim; however, your name and any other identifying information will be excluded from the transcript. Direct quotations from your interview will only be used with your permission and we will contact you later to obtain that permission should we wish to use one of your direct but de-identified quotes.

Please know that you have the right to refuse to answer any questions that make you uncomfortable. If, at any point during the interview, you wish to end the interview, please let me know and we will stop immediately. If you wish to stop the interview, you will have the choice to permit me to retain the interview recording up to that point or to have the interview recording destroyed.

Do you agree to continue with the interview? If so, please sign the document entitled Consent Form to Participate in a Research Study – Community pharmacist champion. I will be documenting your verbal consent for this interview, on my notes and in the recording. Please bring a signed copy of this form and return it to a member of the research team at the Stage 2 meeting.

Do you have any questions before we begin?

Section 1: Regular Follow up and Monitoring Practice

1. Please describe how regular follow up and monitoring of patients happens in your pharmacy.

Section 2: Barriers/facilitators/evaluation criteria

2. What do you think is the most substantial barrier to incorporating routine monitoring and follow up activities in your community pharmacy for people with diabetes?
3. What do you think is the most helpful facilitator to incorporating routine monitoring and follow up activities in your community pharmacy for people with diabetes?
4. What do you think the best opportunity is to link routine monitoring and follow up activities to expanded professional pharmacy services in your community pharmacy for people with diabetes?

Section 3: Outcomes and Strategies

5. What do you think are the most important outcomes to measure to evaluate the success of strategies to improve routing monitoring and follow up for people with diabetes in community pharmacist practice?

Participant Demographics

Do you conduct diabetes medication reviews?

How many diabetes medication reviews does **your pharmacy** typically do on a monthly basis?

How many diabetes medication reviews have **you personally** conducted in the past month?

How much time does it typically take you to perform a diabetes medication review (including preparation time, interview time, and documentation time)?

What is your age in years?

What year did you obtain your first pharmacy professional degree?

What is your pharmacy-related education and/or training?

Are you a Certified Diabetes Educator?

How many years have you worked as a pharmacist in Canada?

What type of community pharmacy do you work at (independent, chain, banner, franchise etc.)

What is your current position at your MAIN community pharmacy practice site?

How many years have you been working in your current position?

On average, how many hours do you work in community pharmacy each week?

Approximately how many prescriptions does your pharmacy fill each day?

What is the postal code of your pharmacy/main practice site?

Surkic N, et al. Exploring the perspectives and strategies of Ontario community pharmacists to improve routine follow-up for patients with diabetes: a qualitative study. *Can Pharm J (Ott)* 2021;154. DOI: 10.1177/17151635211018479.
